# A quick and robust MHC typing method for free-ranging and captive primate species

**DOI:** 10.1007/s00251-016-0968-0

**Published:** 2017-01-13

**Authors:** N. de Groot, K. Stanbury, A. J. M. de Vos-Rouweler, N. G. de Groot, N. Poirier, G. Blancho, C. de Luna, G. G. M. Doxiadis, R. E. Bontrop

**Affiliations:** 1 Biomedical Primate Research Centre, Department of Comparative Genetics and Refinement, Lange Kleiweg 161, 2288 GJ Rijswijk, The Netherlands; 2 Writtle College, Essex University, Lordship Road, Writtle, Chelmsford, Essex CM1 3RR UK; 3 Institut National de la Sante et de la Recherche Medicale (INSERM) UMR1064, Institut de Transplantation-Urologie-Nephrologie (ITUN), 30 Bd Jean Monnet, 44093 Nantes, France; 4 Department of Theoretical Biology and Bioinformatics, Utrecht University, Padualaan 8, 3584 CH Utrecht, The Netherlands

**Keywords:** Primates, MHC, Microsatellites, Conservation biology, Pre-clinical research

## Abstract

Gene products of the major histocompatibility complex (MHC) of human and non-human primates play a crucial role in adaptive immunity, and most of the relevant genes not only show a high degree of variability (polymorphism) but also copy number variation (CNV) is observed. Due to this diversity, MHC proteins influence the capability of individuals to cope with various pathogens. MHC and/or MHC-linked gene products such as odorant receptor genes are thought to influence mate choice and reproductive success. Therefore, MHC typing of wild and captive primate populations is considered to be useful in conservation biology, which is, however, often hampered by the need of invasive and time-consuming methods. All intact *Mhc-DRB* genes in primates appear to possess a complex and highly divergent microsatellite, DRB-STR. A panel of 154 pedigreed olive baboons (*Papio anubis*) was examined for their *DRB* content by DRB-STR analysis of genomic DNA. Using the same methodology on DNA of feces samples, *DRB* variability of a silvery gibbon population (*Hylobates moloch*) (*N* = 24), an endangered species, could successfully be studied. In both species, length determination of the DRB-STR resulted in the definition of unique genotyping patterns that appeared to be specific for a certain chromosome. Moreover, the different STR lengths were shown to segregate with the allelic variation of the respective gene. The results obtained expand data gained previously on DRB-STR typing in macaques, great apes, and humans and strengthen the conclusion that this protocol is applicable in molecular ecology, conservation biology, and colony management, especially of endangered primate species.

**Electronic supplementary material:**

The online version of this article (doi:10.1007/s00251-016-0968-0) contains supplementary material, which is available to authorized users.

## Introduction

The major histocompatibility complex (MHC) class I and II molecules are cell-surface glycoproteins, which play a central role in immune-related processes as in control or susceptibility to infectious diseases, in transplantation research, and in reproduction immunology by presenting peptides to CD8 and/or CD4 T lymphocytes. The MHC genes/loci encoding these molecules are structured in gene families, the hallmark of which is the allelic and copy number variation (CNV) of most of their genes. Balancing selection exerted mainly by pathogen pressure is believed to represent one of the major forces driving MHC polymorphism and diversity (Bernatchez and Landry 2003; Piertney and Oliver 2006; Spurgin and Richardson 2010). Characterization of polymorphic and polygenic MHC genes is therefore an excellent measurement of genetic health and as such of value in conservation biology, as e.g., management of captive breeding programs for endangered species (Cai et al. 2015; Hans et al. 2015; Oliver and Piertney 2012; Pechouskova et al. 2015). One of the most variable MHC genes in many vertebrate species is the *DRB* gene encoding the beta chain of the class II DR molecule. Therefore, *DRB* typing has often been used as an indication for MHC diversity in various species and for diverse purposes, as e.g., the importance of the MHC for mate choice (Setchell et al. 2013; Setchell et al. 2011), heterozygous advantage testing against heterogenous pathogen pressures (Oliver et al. 2009; Osborne et al. 2015), identification of selective pressures acting on MHC haplotypes (Huchard et al. 2008), pathogen-driven balancing selection (Nishita et al. 2015), selection countering drift to maintain MHC polymorphism (Oliver and Piertney 2012), and understanding genetic variability for the management of captive breeding programs (Cai et al. 2015).

**Fig. 1:**
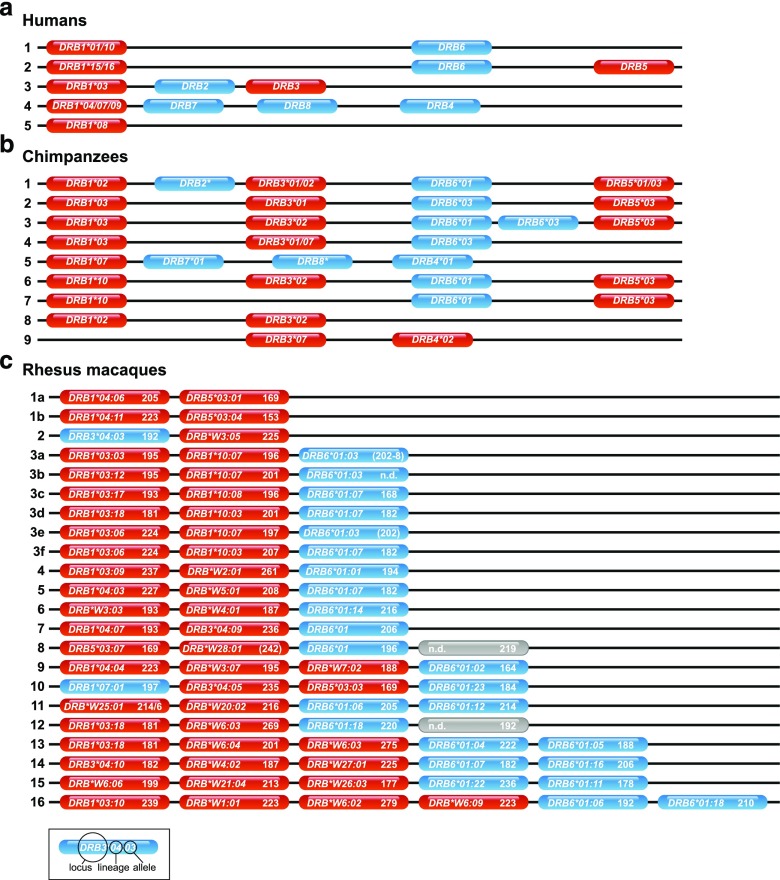
Schematic representation of *DRB* region configurations of **a** humans, **b** chimpanzees, and **c** Indian rhesus macaques. For humans and chimpanzees only, region configurations are presented, whereas for Indian rhesus macaques also, allelic variations are shown accompanied with the observed STR length. *Blue* = pseudogenes, *red* = genes, *gray* = allele not defined (*n*.*d*.)

In primates, the DR region is composed of an utmost oligomorphic *DRA* gene encoding the alpha chain, and polymorphic *DRB* gene(s) encoding one or several beta chains. In humans, for example, five *DR* region configurations have been defined. Each configuration is composed of a *DRA* gene in conjunction with a unique combination of *DRB* genes/lineages, which are usually inherited together as a so-called haplotype. A lineage is defined as group of alleles (allelic variants) within a given gene that cluster together in the phylogenetic tree and are thus supposed to have the same evolutionary origin. The high degree of human *DRB* variability is mainly due to its polymorphic (variable) *DRB1* gene. Chimpanzees, as representatives of the great apes and the closest relatives of humans, show a higher number of region configurations, only three of which displaying allelic variation. This allelic variation is, however, not unique to the *DRB1* gene as polymorphism is present within all genes. In contrast, Old World monkeys (OWM) as macaques show a far higher number of region configurations with a very low degree of allelic variation. This peculiar situation of CNV in OWM is the reason, why *DRB* typing in these animals is hampered by the need of invasive DNA/RNA isolation procedures from blood samples as well as subsequent laborious and expensive techniques such as cloning/Sanger sequencing or next-generation sequencing. However, in close proximity to the highly variable *DRB* exon 2 is a complex microsatellite located in intron 2. This so-called DRB-STR (D6S2878) is present in all primate *DRB* genes analyzed so far with an intact exon 2–intron 2 boundary (Andersson et al. 1987; Bergstrom et al. 1999; Epplen et al. 1997; Riess et al. 1990; Trtkova et al. 1995). In humans, chimpanzees, and macaques, it was recently proven that the length variability of the DRB-STR is in accordance with the allelic variation of the *DRB* gene. Moreover, *DRB* typing with this specific microsatellite resulted in unique genotyping patterns that appear to be specific for a given *DRB* haplotype as illustrated for *DRB* haplotyping in Indian rhesus macaques (see Fig. 1c) (de Groot et al. 2008; Doxiadis et al. 2007; Doxiadis et al. 2013).

Based on these published data, we examined the possibility to type for *DRB* by using the DRB-STR microsatellite in another Old World monkey species, the olive baboon. Very little was known so far about the *DRB* allelic variation and haplotype composition of the olive baboon (*Papio anubis*, *Paan-DRB*), although the olive baboon serves as model species in renal (Poirier et al. 2015), and xeno-transplantation research (Le Bas-Bernardet et al. 2015), and for immunotoxicity studies (Poirier et al. 2014).

An endangered lesser ape species, the silvery gibbon shares a common ancestor with humans ∼18–20 mill years ago (Perelman et al. 2011). The natural habitat of the silvery gibbons is the island of Java, where they occupy lowland and hill and lower mountain rainforests. A population survey of the silvery gibbon carried out between 1994 and 2002 found that 4000 to 4500 individuals were present not only in habitat fragments in the west of Java but also in central Java (Nijman 2004). The rate of which its numbers appear to be declining, mainly due to deforestation, the silvery gibbon has a high chance of going extinct. There are three wild populations located in Java National Parks, and the necessity to improve habitat protection has been recognized (Campbell et al. 2008). The global captive population of silvery gibbons totals 119 individuals, 71 of which are located in zoological institutions in Indonesia, where attempts to breed the species have been largely unsuccessful (Campbell et al. 2008; Cocks 2000). Outside of Indonesia, however, there are 48 individuals spread across several zoological institutions and 24 animals at Port Lympne and Howletts parks within the UK (Stanbury 2015). Port Lympne and Howletts zoos have selected the species for an overall conservation program that includes both a captive breeding and re-introduction program. The International Union for Conservation of Nature (IUCN) recommends that conservation should be carried out at three levels, the ecosystem, the species, and the genetic level (Frankham 2010). For the latter purpose, MHC typing appears to be appropriate, and we therefore wanted to establish the DRB-STR typing method for this endangered species on DNA isolated from feces for animal welfare reasons. So far, nothing was known about the polymorphism and variability of the *DRB* genes of the silvery gibbon (*Hymo-DRB*). Fortunately, primers that had been developed for the amplification of the human DRB-STR could be successfully used for the amplification of *Hymo*- as well as *Paan-DRB*. For the latter, primers used for the amplification of the DRB-STR in macaques could additionally be employed. In such a way, a large cohort of olive baboons, which had been partially pedigreed beforehand, has been defined for their *DRB* content. Although it has been widely reported that both DNA yield and quality derived from fecal samples is lower than that extracted from blood or tissue (Chaves et al. 2006; Marrero et al. 2009; Wasser et al. 1997), feces-extracted DNA of 20 of 21 silvery gibbons, housed at Port Lymphe and Howletts parks, allowed DRB-STR typing as well. *DRB* genotyping and haplotyping results of olive baboons and silvery gibbons will be presented. The data open opportunities for MHC analysis in captured and endangered primates in the context of animal welfare, and the impact on colony management and/or conservation biology will be discussed.

## Material and methods

### Animals

#### Olive baboon

Olive baboons, originally obtained from the Centre National de la Recherche Scientifique Centre de Primatologie (Rousset, France), were housed at the large animal facility of the INSERM unit 1064 (Nantes, France). Blood sampling was performed under anesthesia in accordance with the institutional ethical guidelines. The 154 animals analyzed in this study were partly pedigreed and descended from 34 sires and 105 dames. In most cases, the sires were known but often two males could possibly have sired the offspring.

#### Silvery gibbon

Twenty-four silvery gibbon individuals reside at Port Lympne and Howletts, UK, zoological institutions, of which the pedigree was partly known. Fecal samples were allocated to specific individuals either by direct observation, and two fecal samples per individual were collected immediately or by hand feeding foodstuffs containing maize that could then be identified on subsequent days. Samples were immediately frozen upon collection.

### DNA extraction from EDTA blood of olive baboons

DNA was extracted from fresh EDTA or citrate blood samples using a conventional phenol/chloroform method. Briefly, white blood cells (WBC) were harvested after the lysis of red blood cells and removing the plasma by spinning down the WBCs. WBC pellets were then lysed using a 0.2% SDS and 100 μg/ml proteinase K solution and incubated overnight at 55 °C. An equal volume of phenol was then added to the lysate, and the incubation tubes were intensively mixed and then centrifuged. The upper aqueous layer was harvested and an equal volume of phenol and then of chloroform were added. Tubes were again intensively mixed, centrifuged, and the upper aqueous layer was harvested. Finally, an equal volume of chloroform was added, tubes were mixed, centrifuged, and the upper aqueous layer was harvested again. DNA were precipitated at −20 °C with two volumes of 96% ethanol. The DNA pellets were washed using 70% ethanol and then air-dried before reconstitution in sterile water. DNA concentration were adjusted at 50 ng/μl and stored at 4 °C.

### DNA extraction from fecal samples of the silvery gibbon

DNA was extracted from the frozen fecal samples using the QIAamp DNA stool Mini Kit (Qiagen) following the Stool Larger Volumes protocol. This commercially available kit was chosen as it included a step that involved binding secondary compounds found in plant matter that is present in herbivorous diets, which is applicable to the silvery gibbon species. Plant secondary compounds negatively impact the PCR process by interfering with the *Taq* polymerase enzymatic reaction (Marrero et al. 2009). For each DNA extraction, an amount of 400 mg of frozen stool was used ensuring that both internal and external surfaces of the feces were present as a precautionary measure as sloughed epithelial cells may not be homogenously distributed throughout the sample (Piggott MaT 2003).

### DRB-STR genotyping

Amplification of the relevant DNA segment in olive baboon and silvery gibbon was performed as described for humans and rhesus macaques using the same primer sets (Doxiadis et al. 2007). Briefly, the relevant DNA was amplified with a labeled forward primer located at the end of exon 2, 5′HLA-DRB-STR_VIC and a reverse primer in intron 2, namely 3′HLA-DRB-STR or the unlabeled primer 5′Mamu-DRB-STR together with the labeled macaque specific primer 3′Mamu-DRB-STR_VIC. The labeled primers were synthesized by Applied Biosystems (Foster City, USA) and the unlabeled primers by Invitrogen (Paisley, Scotland). The PCR reaction was performed in a 25-μl reaction volume containing 1 unit of *Taq* polymerase (Invitrogen, Paisley, Scotland) with 0.1 μM of the forward primer, 0.1 μM of the reverse primer, 2.5 mM MgCl_2_, 0.2 mM of each dNTP, 1× PCR buffer II (Invitrogen, Paisley, Scotland), and 100 ng DNA.

The cycling parameters were a 5-min 94 °C initial denaturation step, followed by 5 cycles of 1 min at 94 °C, 45 s at 58 °C, and 45 s at 72 °C. Then the program was followed by 25 cycles with 45 s at 94 °C, 30 s at 58 °C, and 45 s at 72 °C. A final extension step was performed at 72 °C for 30 min. The amplified DNA was prepared for genotyping according to the manufacturer’s guidelines using the GeneScan™ 350Rox™ Size Standard and analyzed on an ABI 3130 genetic analyzer (Applied Biosystems). STR analysis was performed with the Genemapper software 5 program (Applied Biosystems) and all samples were at least analyzed twice. For *Paan-DRB* genotyping both primer pairs, HLA-DRB-STR and Mamu-DRB-STR were used. STRs defined by the HLA primers are named STR-H, and those detected by the macaque primers are called STR-M. Allele bins are defined beforehand.

### PCR, cloning, and sequencing

Forty-four different *Paan-DRB* alleles and 15 different *Hymo-DRB* alleles were sequenced from exon 2 to intron 2, including the microsatellite. Therefore, we used the same primers and PCR reaction as described for humans and rhesus macaques (Doxiadis et al. 2007), and at least 48 clones per animal have been picked and sequenced. All the gained *Paan-DRB* and *Hymo-DRB* sequences were unreported and have been deposited in the EMBL database. In addition the sequences are officially designated by the IPD/MHC database (de Groot et al. 2012; Ellis et al. 2006; Robinson et al. 2003).

## Phylogenetic analyses

Multiple sequence alignments of exon 2 of silvery gibbon *DRB* sequences together with some *DRB* sequences from humans and chimpanzees were performed using MacVector™ version 12.7.5 (Oxford Molecular Group) and phylogenetic analyses were then performed using MEGA version 4.0.2. Pairwise distances were calculated using maximum likelihood and Kimura-2 parameter for creating a phylogram. Confidence estimates of grouping were calculated according to the bootstrap method generated from 1000 replicates and the tree was rooted with Caja-DRB*W16:01.

## Data accessibility

DNA sequences are deposited to the EMBL gene bank with accessions numbers JQ666205–JQ666210, JQ666212–JQ666215, JQ666217–JQ666230, JQ666232–JQ666251, KJ701253–KJ701266, and LN867601).

## Results

### Paan-DRB-STR typing

**Fig. 2:**
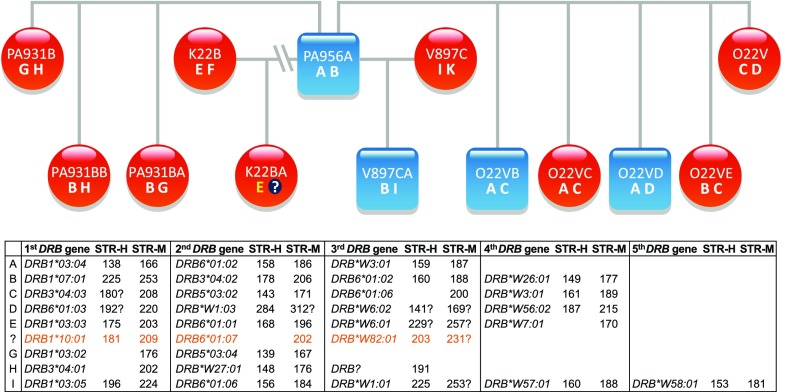
Segregation of *DRB* haplotypes (A–I) within an olive baboon family. The *DRB* alleles and their adjacent DRB-STR lengths, which define haplotypes A–I, are given below. *Blue squares* = males, *red circles* = females, *question mark* = *DRB* haplotype defined in animal K22BA, which could not be detected in sire PA956A

A large cohort of olive baboons (# 154) of mostly two generations originating from 34 sires and 105 dames have been analyzed with the DRB-STR method. As has been observed for other primates, microsatellite typing of the baboon samples with this STR resulted in the definition of various microsatellite patterns. As comparison, Fig. 1 shows the *DRB* region configurations of humans (Fig. 1a), chimpanzees (Fig. 1b), and Indian rhesus macaques (Fig. 1c), the latter of which the DRB-STR patterns are given in addition. Depending on the animal, the number of the different STR lengths in olive baboons varied from four to ten. According to co-segregation of certain microsatellite lengths within a family, patterns can be deduced which appear to represent *DRB* haplotypes. As has been shown in earlier studies for other primate species, each *DRB* allele has its own characteristic microsatellite length. Sequencing of *DRB* exon 2 together with the microsatellite-containing intron 2 verified that indeed each *DRB* allele is accompanied by a specific STR length. An example of such a *DRB* haplotype distribution within a baboon family is shown in the pedigree of parts of a large baboon family, and nine different haplotypes could be defined with numbers of *DRB* loci/genes per haplotype varying from three to five (Fig. 2, haplotypes A–I). In most cases, the DRB-STR could be defined with the macaque primers (Fig. 2, STR-M), but sometimes amplification was successful using the human primers instead (Fig. 2, STR-H). All *DRB* genes could be identified unambiguously by sequencing with one exception only (Fig. 2 haplotype H, STR-H 191). Likewise, there was one *DRB* allele, of which the STR could not be determined (Fig. 2, haplotype D, DRB*W6:02). These infrequent amplification failures of DRB alleles or STR sequences are most probably due to primer inconsistencies. Although dames and sires of the first generation were mostly not available for the analyses, it can be shown that sire PA956A has not fathered offspring K22BA, since this animal does not share one of the sire’s *DRB* haplotypes.

**Fig. 3:**
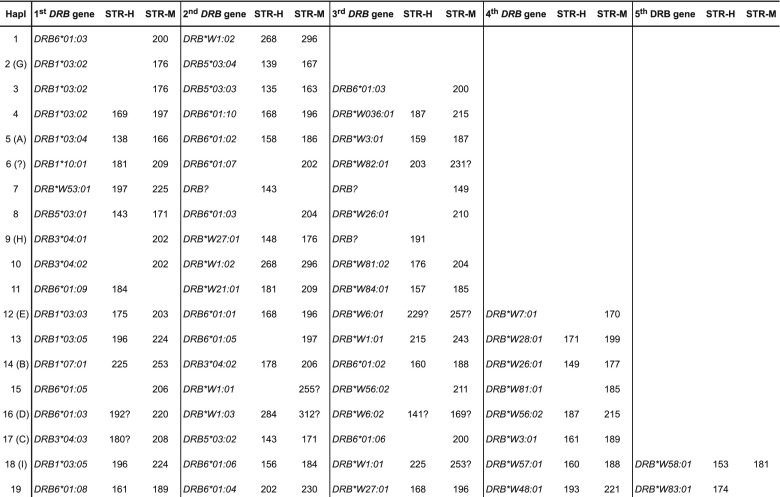
*Paan-DRB* haplotypes defined by exon 2 sequencing and DRB-STR genotyping. *Question marks* indicate that STR is not or rarely detected but presence of allele is ascertained by sequencing

Based on the *DRB* typing results of the entire olive baboon panel, we have been able to define 19 different *DRB* region configurations/haplotypes varying in content and numbers of *DRB* genes present (Fig. 3). Configurations/haplotypes that have already been observed in the baboon family (Fig. 2) are indicated by the corresponding letters (e.g., Hapl. 2 in Fig. 3 is the same as Hapl. G in Fig. 2). No allelic variation within a given region configuration could be detected. Together with five *DRB* alleles that have been observed in a single haplotype only and are therefore not included in Fig. 2, a total of 50 *Paan-DRB* alleles have been newly defined (Suppl. Table 1), which belong to a high variety of lineages, and amino acid sequences have been deduced (Suppl. Table 2). Eleven of the *Paan-DRB* sequences are identical to a *DRB* allele of the Chacma baboon (*Paur-DRB*), the only baboon species of which many *DRB* alleles had been sequenced in the past (Huchard et al. 2008) (Suppl. Table 3). This sharing of *DRB* alleles within the genus *Papio* has been expected due to the trans-specific mode of evolution (Klein et al. 1993; Perelman et al. 2011). As has been observed in all apes and Old World monkey species studied so far, the number of *DRB* genes per haplotype may vary, in the olive baboon between two to five. Additionally to the one *DRB* allele of the baboon family, which could not be defined (Fig. 2, haplotype H, STR-H 191, STR-M 215), two other *DRB* alleles could not be determined although STRs have been identified (Fig. 3, haplotype 7). As discussed above, the rare failure to amplify certain alleles is most probably due to primer inconsistencies. As in macaques, which nearly show any allelic variation within region configurations, no allelic variation can be observed at all within a certain *Paan-DRB* region configuration, and thus, the number of *Paan-DRB* region configurations is identical to the number of different *DRB* haplotypes. As has been shown for other primate species, each *Paan*-*DRB* haplotype has its own characteristic DRB-STR pattern that co-segregates within a family.

### Hymo-DRB-STR typing

**Fig. 4:**
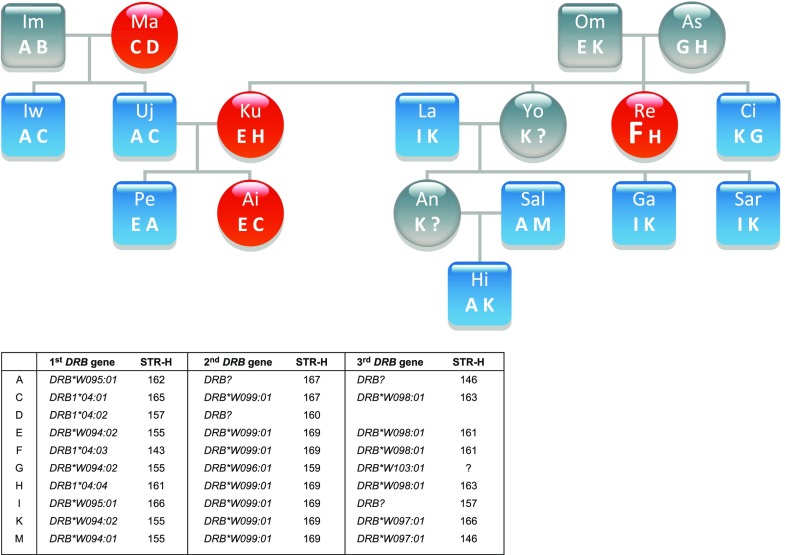
Segregation of *DRB* haplotypes (A–M) within a silvery gibbon family. The *DRB* alleles and their adjacent DRB-STR lengths, which define haplotypes A–M, are given below. *Blue squares* = males, *red circles* = females, *question mark* = *DRB* haplotype that could not been defined, *F* = haplotype of female Re which is not present in sire Om

**Fig. 5:**
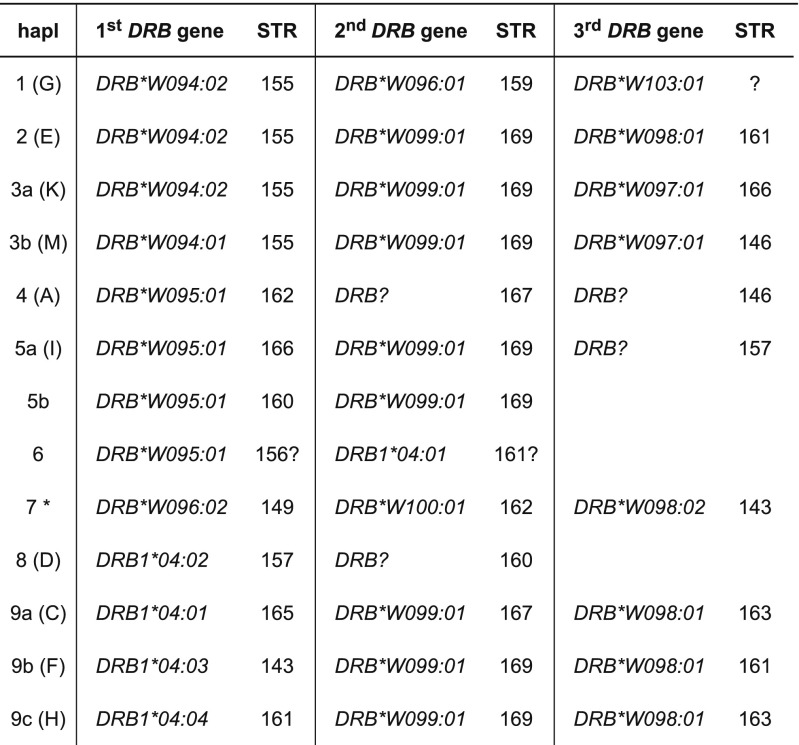
*Hymo-DRB* haplotypes defined by exon 2 sequencing and DRB-STR genotyping. *Asterisk* = haplotype not certain. DRB-STR 161? is detected but could not be confirmed

**Fig. 6:**
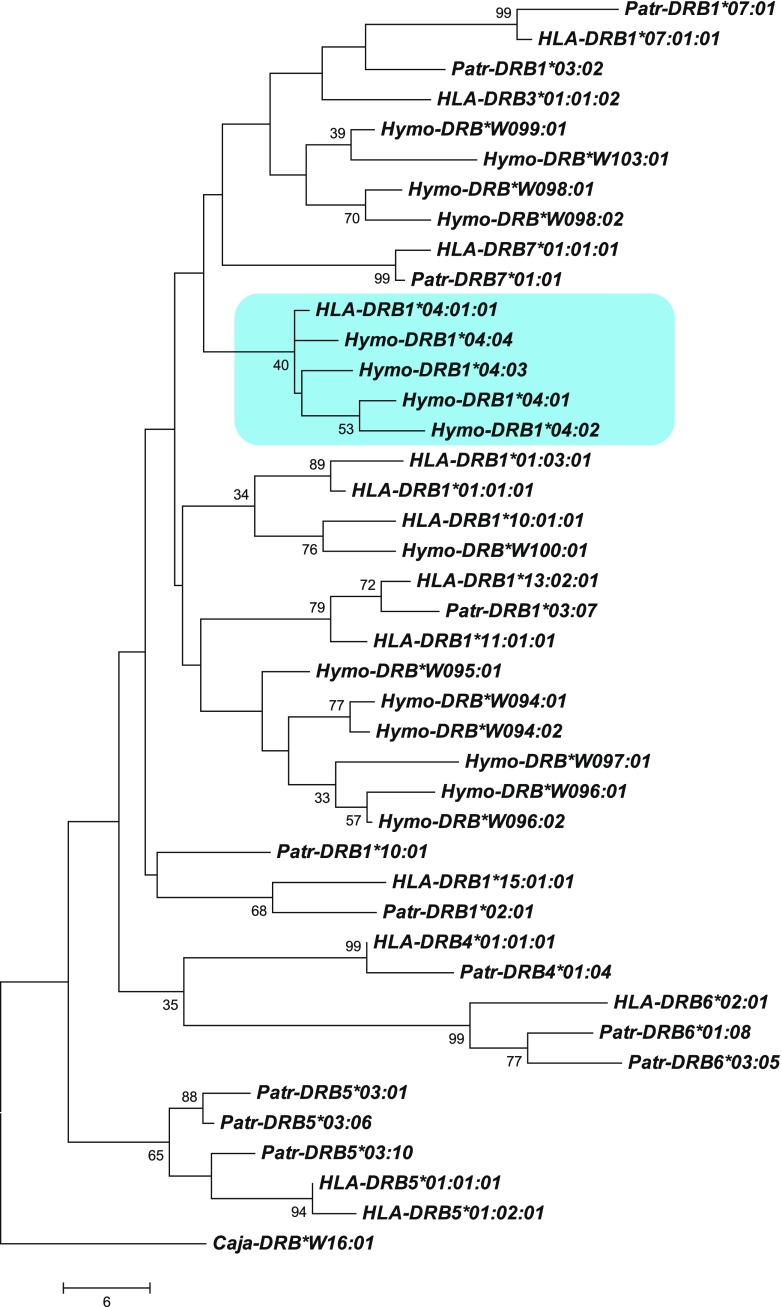
Phylogenetic tree of *DRB* exon 2 sequences of humans (*HLA-DRB*), chimpanzees (*Patr-DRB*), and silvery gibbons (*Hymo-DRB*)

From the silvery gibbon studbook managed by Howletts zoo (Stanbury 2015) pedigree data of the families, founded by two silvery gibbon pairs and one additional female and male, were partly present for the 21 individuals of which DNA has been collected. To type for *DRB* of the silvery gibbon (*Hymo-DRB*) the same approach as for the olive baboon was followed. With the human DRB-STR primers, the *DRB* microsatellites could be successfully amplified of all but one silvery gibbon. As for the olive baboons, the amplification resulted in specific microsatellite patterns, which segregate in a Mendelian fashion. Subsequent sequencing of exon 2 plus the adjacent microsatellite showed that also in silvery gibbons, a certain microsatellite length is indicative for a *DRB* allele, and a *DRB* haplotype can be defined by a specific microsatellite pattern. As a result, part of the pedigree of the two founder pairs can be substantiated by the segregation of *DRB* haplotypes (Fig. 4, haplotypes A–M). The female child Re, however, is most probably not fathered by sire Om, since she does not share a haplotype with this male. The analysis of *DRB* of all animals resulted in the definition of nine region configurations (Fig. 5; region configurations, which had already been defined in the silvery gibbon’s pedigree, are indicated by letters next to haplotype numbers). Although we cannot rule out the possibility that we did not detect all *DRB* alleles by analyzing DNA isolated from feces, the number of *DRB* alleles (*N* = 15) and the composition of the *DRB* region configurations/haplotypes appears to show a low degree of variability. In contrast to other primate species and especially to Old World monkeys as baboons (Fig. 3), most *Hymo*-*DRB* haplotypes seem to be constructed by three *DRB* loci. In addition, the *DRB* alleles belong to only a small number of *DRB* lineages but show instead allelic variation within a certain region configuration. As a consequence, 13 different *DRB* haplotypes could be determined, most of which consist of combinations of alleles of the same, few lineages (Fig. 5, e.g., DRB1*04; DRB*W094, DRB*W098). Furthermore, the lineage DRB*W099 seems to be monomorphic and its one allele, DRB*W099:01, is present on 8 of 13 haplotypes. Remarkably, the *DRB5* and *DRB6* lineages, which are both old entities in primate evolution, are not observed (Doxiadis et al. 2012). However, we cannot exclude that those alleles, which could not be amplified with the primers used (Fig. 5, “?”), probably belong to these lineages. Phylogenetic analysis of exon 2 of *Hymo-DRB* alleles together with a selection of human and chimpanzee *DRB* alleles shows that the silvery gibbon alleles defined in this study cluster into two species-specific branches with two exceptions; first, Hymo-DRB*W100:01 groups apart together with a human DRB1*10 lineage member; the second exception concerns the Hymo-DRB1*04 alleles which cluster together with HLA-DRB1*04:01:01 (Fig. 6). From previous studies, we know that the HLA-DRB1*04 lineage is absent in the chimpanzee and probably also in other ape species and represents together with HLA/Patr-DRB1*07 and HLA-DRB1*09 an evolutionarily old entity (Doxiadis et al. 2012). It is therefore remarkable that the DRB1*04 lineage is retained in silvery gibbons, an observation, which is confirmed by an identical motif within the peptide binding groove (Suppl. Fig. 1, amino acids EQVKH, turquois) in silvery gibbons, humans, and even in rhesus macaques. The intron 2 microsatellite sequences of Hymo-DRB1*04 alleles, however, are different from the human DRB1*04 sequence (Suppl. Fig. 2) indicating that intron sequences may vary between species.

## Discussion

In two non-human primate species, for which no MHC class II data have been available so far, the analysis of the *Mhc-DRB* allele content has been effectively performed by the presented robust, time-saving, and inexpensive microsatellite typing technique. In such a way, the *DRB* repertoire of an olive baboon population could be defined. The results obtained are of importance for biomedical research as in transplantation (Le Bas-Bernardet et al. 2015; Poirier et al. 2015), for which these animals are a model species. Due to the high CNV of the *DRB* genes in baboons and the possibility to define *DRB* haplotypes, the *DRB* content in these animals can be followed in addition for colony management reasons as parentage testing and calculation of the inbreeding coefficient.

As has been shown previously for a wild Assamese macaque population (Muller et al. 2014), DRB-STR typing was also successful when DNA isolated from non-invasively collected fecal material was used. This is especially important in the case of wild or free ranging endangered species such as the silvery gibbon. The quality of the isolated DNA was high, most probably due to the immediate freezing of the sample. As a consequence, cloning and sequencing of *DRB* exon 2 including the microsatellite was feasible, and in such a way the DRB-STR length could be linked to a certain *DRB* allele. Therefore, we were able to get an insight into the *DRB* repertoire of the silvery gibbon, which shows an important reduction in the number of lineages present and in CNV in comparison to chimpanzees, humans, and especially to the olive baboon analyzed. Due to the existence of several, mostly three, *DRB* genes with adjacent microsatellites per haplotype, *DRB* haplotypes could be defined for the silvery gibbon, too. Although the genetic repertoire as defined for *Hymo-DRB* appears to be reduced, we were able to confirm the pedigree of two related founder families as recorded in the studbook. In addition, results with this one microsatellite indicated that the father of one of the animals was incorrectly assigned.

Despite their conservation importance, distinct characteristics and high species diversity gibbons are mostly neglected by population genetic studies (Kim et al. 2011). However, several reports describe the high rates of chromosomal rearrangements that can be observed not only in the large number of rearrangements separating gibbons from other apes and humans (Carbone et al. 2006; Muller et al. 2014) but also the numerous rearrangements that separate different gibbon species from each other as well as rearrangements that are polymorphic within a species (Carbone et al. 2009; Van Tuinen et al. 1999). The possible mechanism for this genomic plasticity may be a gibbon-specific retrotransposon, LAVA (Carbone et al. 2014). The observed “patchwork” pattern of most of the *DRB* haplotypes of the silvery gibbon, which are built up of three *DRB* genes of only a few lineages, may also be the result of a near-instantaneous radiation (Carbone et al. 2014) and rearrangements in the *DRB* region of this species.

The complex microsatellite in *DRB* intron 2 is an evolutionarily old entity, which has been observed not only in apes and OWM but also in Platyrrhini species as in marmosets and owl monkeys (Trtkova et al. 1995), and it is observed even in sheep (Ballingall et al. 2008). In the two *Aotus* species it is highly polymorphic and has been used for *DRB* typing, too (Lopez et al. 2014). Thus, this typing method is applicable for nearly all primates. Since DRB-STR typing is highly informative and can additionally be easily performed by feces collection, this methodology can be used to observe differences in genetic fitness between wild and free-ranging animals. Additionally, this method is applicable for monitoring, e.g., reproduction, parentage typing, mate choice, parasite susceptibility, and conservation. Furthermore, in the case of breeding of non-human primates, the *DRB* microsatellite is valuable for parentage typing, avoidance of inbreeding and genetic reduction, and thus for colony management in general.

## Electronic supplementary material

ESM 1 (PDF 71 kb)

ESM 2 (PDF 32 kb)

ESM 3 (PDF 32 kb)

Suppl. Fig. 1 Alignment of deduced amino acids of part of *DRB* exon 2 of humans, chimpanzees, rhesus macaques, and silvery gibbons. The alignment is sorted according the amino acids (9–13) of the peptide binding site. (PDF 27 kb)

Suppl. Fig. 2 Nucleotide composition of the DRB-STR in intron 2 of humans, chimpanzees, and silvery gibbons (PDF 54 kb)
