# Supplementary material for: A quick and robust MHC typing method for free-ranging and captive primate species
Source: Immunogenetics. 2017 Jan 13;69(4):231–40. doi: 10.1007/s00251-016-0968-0 (PMC5350218; doi:10.1007/s00251-016-0968-0)
Supplement: Supplementary file 1 — (PDF 71 kb) [file 251_2016_968_MOESM1_ESM.pdf]

Suppl. Table 1. Accession/IPD numbers of new *Paan*- and *Hymo-DRB* alleles

#	Designation	Accession Number	IPD
1	*Paan-DRB*W21:01*	JQ666205	70007815
2	*Paan-DRB*W81:01*	JQ666206	70007816
3	*Paan-DRB*W6:01*	JQ666207	70007817
4	*Paan-DRB*W6:02*	JQ666208	70007818
5	*Paan-DRB*W7:01*	JQ666209	70007819
6	*Paan-DRB*W81:02*	JQ666210	70007820
7	*Paan-DRB*W81:03*	JQ666211	70007821
8	*Paan-DRB*W1:01*	JQ666212	70007822
9	*Paan-DRB*W1:02*	JQ666213	70007823
10	*Paan-DRB*W1:03*	JQ666214	70007824
11	*Paan-DRB*W3:01*	JQ666215	70007825
12	*Paan-DRB*W4:01*	JQ666216	70007826
13	*Paan-DRB*W27:01*	JQ666217	70007827
14	*Paan-DRB*W28:01*	JQ666218	70007828
15	*Paan-DRB*W48:01*	JQ666219	70007829
16	*Paan-DRB*W53:01*	JQ666220	70007830
17	*Paan-DRB*W82:01*	JQ666221	70007831
18	*Paan-DRB*W56:01*	JQ666222	70007832
19	*Paan-DRB3*04:01*	JQ666223	70007833
20	*Paan-DRB3*04:02*	JQ666224	70007834
21	*Paan-DRB*W56:02*	JQ666225	70007835
22	*Paan-DRB3*04:03*	JQ666226	70007836
23	*Paan-DRB*W26:01*	JQ666227	70007837
24	*Paan-DRB*W57:01*	JQ666228	70007838
25	*Paan-DRB*W83:01*	JQ666229	70007839
26	*Paan-DRB*W58:01*	JQ666230	70007840
27	*Paan-DRB1*03:01*	JQ666231	70007841
28	*Paan-DRB1*07:01*	JQ666232	70007842
29	*Paan-DRB1*10:01*	JQ666233	70007843
30	*Paan-DRB*W84:01*	JQ666234	70007844
31	*Paan-DRB1*03:02*	JQ666235	70007845
32	*Paan-DRB1*03:03*	JQ666236	70007846
33	*Paan-DRB1*03:04*	JQ666237	70007847
34	*Paan-DRB1*03:05*	JQ666238	70007848
35	*Paan-DRB5*03:01*	JQ666239	70007849
36	*Paan-DRB5*03:02*	JQ666240	70007850
37	*Paan-DRB5*03:03*	JQ666241	70007851
38	*Paan-DRB5*03:04*	JQ666242	70007852
39	*Paan-DRB6*01:01*	JQ666243	70007853
40	*Paan-DRB6*01:02*	JQ666244	70007854
41	*Paan-DRB6*01:03*	JQ666245	70007855
42	*Paan-DRB6*01:04*	JQ666246	70007856
43	*Paan-DRB6*01:05*	JQ666247	70007857
44	*Paan-DRB6*01:06*	JQ666248	70007858
45	*Paan-DRB6*01:07*	JQ666249	70007859
46	*Paan-DRB6*01:08*	JQ666250	70007860
47	*Paan-DRB6*01:09*	JQ666251	70007861
48	*Paan-DRB*W036:01*	KX065446	70014381
49	*Paan-DRB6*01:10*	KX065447	70014383

#	Designation	Accession Number	IPD
1	*Hymo-DRB*W094:01*	KJ701253	70011945
2	*Hymo-DRB*W094:02*	KJ701254	70011947
3	*Hymo-DRB*W095:01*	KJ701255	70011949
4	*Hymo-DRB*W096:01*	KJ701256	70011951
5	*Hymo-DRB*W096:02*	KJ701257	70011953
6	*Hymo-DRB1*04:01*	KJ701258	70011955
7	*Hymo-DRB1*04:02*	KJ701259	70011957
8	*Hymo-DRB1*04:03*	KJ701260	70011959
9	*Hymo-DRB1*04:04*	KJ701261	70011961
10	*Hymo-DRB*W097:01*	KJ701262	70011963
11	*Hymo-DRB*W100:01*	KJ701263	70011965
12	*Hymo-DRB*W098:01*	KJ701264	70011967
13	*Hymo-DRB*W098:02*	KJ701265	70011969
14	*Hymo-DRB*W099:01*	KJ701266	70011971
15	*Hymo-DRB*W103:01*	LN867601	70011973
