# Supplementary material for: A quick and robust MHC typing method for free-ranging and captive primate species
Source: Immunogenetics. 2017 Jan 13;69(4):231–40. doi: 10.1007/s00251-016-0968-0 (PMC5350218; doi:10.1007/s00251-016-0968-0)
Supplement: Supplementary file 2 — (PDF 32 kb) [file 251_2016_968_MOESM2_ESM.pdf]

Suppl. Table 2. Deduced amino acid sequences of Paan-DRB exon 2 as defined in this study

HLA-DRB1*01:01:01	LWQLKFECHFFNGTERVRLLERCIYNQEESVRFDSDVGEYRAVTELGRPDAEYWNSQKDLLEQRRAAVDTYCRHNYGVGESFTVQRR
Paan-DRB1*03:01	-EYSTS------------F-D-YF-----N---------F---S-----------GR--I--DE--S---F-----R----------
Paan-DRB1*03:02	-EYSTS-------M----F-D-YF-----Y---------F---S----RS------R--Y--DE---------Y----V--------
Paan-DRB1*03:03	-EYSTS------------F-D-YF-----Y---------F---S----RS------R--Y--DE---------Y----V--------
Paan-DRB1*03:04	-EYSTS------------F-D-YF-----L-------------S-----E--S---R--V--RA-T---N---Y--R----------
Paan-DRB1*03:05	-EYSTS------------Y-D-YF-----Y---------------------------------K-GQ--N-----------------
Paan-DRB1*07:01	---S-AK-----------Y---H------LM------------S-----A------L--Y---K--------------V--------
Paan-DRB1*10:01	-EYV--------------Y-I-VFH-R--YA--------F--------RS---------F---K--------------V--------
Paan-DRB3*04:01	-E-G-S------------Y-Q-HF-----F---------F---------V--S------Y-------------Y--R----------
Paan-DRB3*04:02	-E-G-S------------Y-Q-HF-----F---------F---------V--S------Y---------NF--Y--Q----------
Paan-DRB3*04:03	-E-G-S------------Y-Q-HF-----F---------F---------V--S------Y-------------Y--Q----------
Paan-DRB5*03:01	-K-D-Y--------------H-Y------DA----------------------------V------E---V-----RGV--------
Paan-DRB5*03:02	-K-D-Y--------------H-D------DA------------------V---L-----V------E---V-----RGV--------
Paan-DRB5*03:03	-K-E----------------H-Y------D--V---I----
Paan-DRB5*03:04	-K-D--------------F-H-Y------DA-------------------------R--V--D---Q---V-------V--------
Paan-DRB6*01:08	-E-G-S--QI---R----Y-N-N-HKR--NL--H-----FQ--M-----V--N-----GI--EK-DK------Y--R-F-------*
Paan-DRB6*01:09	-E-G-S--QI---R-P--Y-N-N-HKR-GNL--------FQ--------V--N-----GI--EK-DK------Y--R-F----E--*
Paan-DRB6*01:01	-E-A-C---I-------QY-D-Y-HKR--NL--H-----FQ--------V--N-----GI--*K-DK--S---Y----F----E--*
Paan-DRB6*01:04	-E-A-CK--I-----P-QY-N-Y-HKR--NL--H-----FQ-----EW-V--N-----GI--*K-DK--S---Y----F----E--*
Paan-DRB6*01:10	-E-A-C---I-----Q-QY-D-Y-HKR--NL--H-----FQ--------V--N-----GI--*K-DK--S---Y----F----E--*
Paan-DRB6*01:02	-E-A-C---I--------Y-N-N-HKR--NL--H--L--FQ-----E--V--N-----GI--EK-DK------Y--R-F----E--*
Paan-DRB6*01:05	-E-A-C---I-------QYPN-N-HKR--NL--H--L--FQ-A-Q----V--N-----GI--EK-DK------Y----F----E-P-
Paan-DRB6*01:07	-E-A-C--PI-------QY-N-Y--KR--NL--H--L--FQ---Q----V--N-----GI--EK-D*------N----F----E--*
Paan-DRB6*01:03	-E-A-C---I------MQY-N-Y-HKR--NL--H--L--FQV-------V--N-----GI--EK-D*-----KY--R-F----E--*
Paan-DRB6*01:06	-E-A-C---I-------QY-N-Y-HKR--NL--H--L--FQ--------V--N----NGI--EK-D*-A----Y----F----EH--
Paan-DRB*W1:01	-EYC-----------L-LY-I-YFH----Y--Y----------------V--N-------------E--N-----------------
Paan-DRB*W1:02	-EYC-------------LN-I-YF-----Y--Y---------------------------------E--------------------
Paan-DRB*W1:03	-EYC-------------LN-I-YF-----Y--Y---------------------------------E-----------V--------
Paan-DRB*W3:01	-E-A-R------------F-D-YFH----YA--------F--------RS---F-----F---A-T---N---Y-------------
Paan-DRB*W4:01	-EHV-S------------F---HF-----NL------------------E--S------I--EK--R--N------R----------
Paan-DRB*W6:01	-K-T-A--------------D-HF-----Y--Y--Y------------RS---F-----Y--RT--E---------R-V--------
Paan-DRB*W6:02	-K-T-A--------------D-HF-----Y--Y--Y------------RS---F-----Y----------------R----------
Paan-DRB*W7:01	-Q-Q-A--R---------F---Y------Y------------------RS---------A--EK--R---------R----------
Paan-DRB*W21:01	-E-V-S------------F---YF-----Y-------------S----LS-----G-------K-GQ--N--------V--------
Paan-DRB*W26:01	-E-A-S------------F---HFH----YA--------F---------A--S------Y--DT--S---F--Y-------------
Paan-DRB*W27:01	-E-A-S------------Y-D-Y-H----F---------H----------------R--I--DQ------F--Y--R-F--------
Paan-DRB*W28:01	---A-R------------F-D-Y------LL--------F---------T--N---RQ-V---A-----N--------V--------
Paan-DRB*W036:01	FEYCTH------------Y-V-FF--R--Y----N----FQ--------E--S----------KV--E--IF------V--------
Paan-DRB*W48:01	-E-G-S------------F---YFH----F----------------------S---R------K-GQ--N---Y----V-N------
Paan-DRB*W53:01	-E-H-A--------------D-Y-H----FL--------F---------V--NL-TR-E---RE--Q---V------GV--------
Paan-DRB*W56:01	-Q-F-S------------Y-Q-HF-----F---------F---------V---L-----Y------Q--N---Y--R----------
Paan-DRB*W56:02	-E-F-S-----D------Y-Q-HF-----F-------------------A---L-----Y------QA-N--------F--------
Paan-DRB*W57:01	-E-A-S------------Y-D-YF-----Y---------F-----------Q-------F--DS-----------------------
Paan-DRB*W58:01	-QRDYL-----------QY---YF-----FL----H---F---S-----E--S---R--V--D---R--*I--R--R-V--------
Paan-DRB*W81:01	---F-P------------F-D-YF-----F---------F-----------Q-------I--DE------F------GV--------
Paan-DRB*W81:02	---F-P------------F-D-YF-----LL------------S-----Q--S------F--D---Q---V-----R-V--------
Paan-DRB*W81:03	---F-P------------F---HF-----LL------------S-----Q--S------F--D---Q---V-----R-V--------
Paan-DRB*W82:01	-E-V-H------------F-D-YF-----YA-------------------------------R---E--------------------
Paan-DRB*W83:01	-E-A-S------------F-D-Y-H----FA--------F------------S---------G-------------RIA--------
Paan-DRB*W84:01	-E-A-C------------Y---YM-----YA--------F------------N---R--V--D---R--IF----------------
