# Supplementary material for: A quick and robust MHC typing method for free-ranging and captive primate species
Source: Immunogenetics. 2017 Jan 13;69(4):231–40. doi: 10.1007/s00251-016-0968-0 (PMC5350218; doi:10.1007/s00251-016-0968-0)
Supplement: Supplementary file 3 — (PDF 32 kb) [file 251_2016_968_MOESM3_ESM.pdf]

Suppl. Table 3. *Paan-DRB* alleles identical to *Paur-DRB* alleles

*Paur-DRB1*0301*	*Paan-DRB1*03:02*
*Paur-DRB5*0302*	*Paan-DRB5*03:04*
*Paur-DRB1*0302*	*Paan-DRB1*03:04*
*Paur-DRB6*0101*	*Paan-DRB6*01:01*
*Paur-DRB*W301*	*Paan-DRB*W3:01*
*Paur-DRB*W101*	*Paan-DRB*W1:01*
*Paur-DRB6*0103*	*Paan-DRB6*01:05*
*Paur-DRB*W2801*	*Paan-DRB*W28:01*
*Paur-DRB*W2701*	*Paan-DRB*W27:01*
*Paur-DRB5*0301*	*Paan-DRB5*03:01*
*Paur-DRB*W5601*	*Paan-DRB*W56:01*
