# Supplementary material for: A quick and robust MHC typing method for free-ranging and captive primate species
Source: Immunogenetics. 2017 Jan 13;69(4):231–40. doi: 10.1007/s00251-016-0968-0 (PMC5350218; doi:10.1007/s00251-016-0968-0)
Supplement: Supplementary file 4 — Alignment of deduced amino acids of part of DRB exon 2 of humans, chimpanzees, rhesus macaques, and silvery gibbons. The alignment is sorted according the amino acids (9–13) of the peptide binding site. (PDF 27 kb) [file 251_2016_968_MOESM4_ESM.pdf]

```
HLA- DRB1*01:01:01    RFLWQLKFECHFFNGTERVRLLERCIYNQEESVRFDSDVGEYRAVTELGRPDAEYWNSQKDLLEQRRAAVDTYCRHNYGVGESFTVQRR
HLA- DRB5*01:01:01    ---Q-D-Y------------F-H-D------DL----------------------------F--D------------------------
HLA- DRB5*01:02:01    ---Q-D-Y------------F-H-G------N-----------------------------F--D------------------------
Patr-DRB5*03:10       ---K-D-Y------------F-H-YF-----DL----------------------------I--R---E--------------------
Patr-DRB5*03:01       ---K-D-Y------------F-H-G------DL----------------------------I--R---D---F-------L--------
Patr-DRB5*03:06       ---K-D-C------------F-H-D------DL----------------------------I--R---------------L--------
HLA- DRB1*01:03:01    -------------------------------------------------------------I--DE-----------------------
HLA- DRB1*01:01:01    -----------------------------------------------------------------------------------------
Patr-DRB1*10:01       ---E-A-C------------F---R-H----D-----------------------------I-A----D---F----------------
HLA- DRB1*10:01:01    ---EEV------------------RVH----YA-Y-----------------------------R------------------------
Hymo-DRB*W100:01      ---E-V-Y-----------Q----RVH----YA----------------------------I------E---V----------------
Patr-DRB1*02:01       ---L-P-G------------F---D------FM------------------V---C-----I---A-----N-----------------
HLA- DRB1*15:01:01    -----P-R------------F-D-YF---------------F-------------------I---A--------------V--------
HLA- DRB7*01:01:01    ---E-A-S----------_LY-*-YF-----Y---N--M--F-------------------I---K--E--N*-------V-R------
Patr-DRB7*01:01       ---E-A-S----------_LY-*-YF-----Y---N--M--F-------------------I---K--E--N--------V-R------
Patr-DRB6*03:05       ---E-A-C---I-------QY-N-Y-HKR--NL------E-FQ------Q-V--N-----GI--EK-DK------Y--R-F*--S---*
Patr-DRB6*01:08       ---EKA-C---I----K--QY-N-Y-HKR--NL------E-FQ--------V--N-----GI--EK-DK--I---Y----F------PS
HLA- DRB6*02:01       ---E-A-C---I---MK--QY-N-Y-HKR--NL----N-E-FQ--------V--N-----GIP-EK-DKM-D---Y----F*ELHSAAA
HLA- DRB4*01:01:01    ---E-A-C----L------WN-I-Y------YA-YN--L---Q---------------------R---E------Y----V--------
Patr-DRB4*01:04       ---E-A-H----L------WN-I-Y------YA-YN--L---Q----------K----------RT--E------Y----V-------*
HLA- DRB3*01:01:02    ---ELR-S------------Y-D-YFH----FL------------------V--S----------K-GR--N-----------------
Hymo-DRB*W101:01      ---E-A-A------------Y-Q-YF-----FL------------------E--N------F---K-GQ--N-----------------
Hymo-DRB*W099:01      ---E-A-S------------F---YF-----Y----------------------N------F---K-GR--N-----------------
Hymo-DRB*W098:01      ---E-A-S------------F---YF-----Y----------W--------S-K-------I---K-GR--N-----------------
Hymo-DRB*W098:02      ---E--NS------------F---YF-----Y----------W--------S-K-------F---K-GQ--N-----------------
Hymo-DRB*W094:01      ---E-A-A----------------HF-----F----------------------N------F------E--N--------V--------
Hymo-DRB*W094:02      ---E-A-A----------------HF-----F----------------------N------F---------N-----------------
Hymo-DRB*W095:01      ---E-A-S------------F-D-Y-H----IL---------------------N------F---------N--------F--------
Hymo-DRB*W097:01      C--E-V-S-------M----F---Y-H----F----------------------N---------RK-GQ--N-------GV--------
Hymo-DRB*W096:01      ---E-G-A------------Y---Y-H----F----------------------N---------EA-----N-----------------
Hymo-DRB*W096:02      ---E-G-A------------Y---Y-H----F----------------------N----------K-----N--------V--------
Patr-DRB1*03:02       ---EYSTS------------F-D-YFH----Y-------------------V--S------I--D--GQ--N-----------------
HLA- DRB1*11:01:01    ---EYSTS------------F-D-YF-----Y---------F----------E--------F--D------------------------
HLA- DRB1*13:02:01    ---EYSTS------------F-D-YFH----N---------F-------------------I--DE-----------------------
Patr-DRB1*03:07       ---EYSTS------------F-D-YFH--------------F-------------------YV-DE--------------A--------
Mamu-DRB1*04:04       ---E-V-H------------F-D-YF-----Y-----------------------------F--D---Q---V-------V--------
Hymo-DRB1*04:04       ---E-V-H------------F-D-YF-----Y----------------------N------F---------N-----------------
Hymo-DRB1*04:03       ---E-V-H------------F-D-YF-----Y---------------------Q----------EA-----N-----------------
Hymo-DRB1*04:01       ---E-V-H------------F-D-YF-----Y---------------------Q-----------K-GR--N--------V--------
Hymo-DRB1*04:02       ---E-V-H------------F-D-YF-----Y-------------------S---------F---K-GR--N-----------------
HLA- DRB1*04:01:01    ---E-V-H------------F-D-YF-H---Y---------------------------------K-----------------------
HLA- DRB1*07:01:01    -----G-YK----------QF---LF-----F-------------------V--S------I--D--GQ---V----------------
Patr-DRB1*07:01       -----S-YK----------QF---LF-----F-------------------V--S---------D--GQ---V-------L---I----
```
