# Supplementary material for: A quick and robust MHC typing method for free-ranging and captive primate species
Source: Immunogenetics. 2017 Jan 13;69(4):231–40. doi: 10.1007/s00251-016-0968-0 (PMC5350218; doi:10.1007/s00251-016-0968-0)
Supplement: Supplementary file 5 — Nucleotide composition of the DRB-STR in intron 2 of humans, chimpanzees, and silvery gibbons (PDF 54 kb) [file 251_2016_968_MOESM5_ESM.pdf]

DRB alleles	Part 1 (GT)n	Part 2 mixed	Part 3 (GA)n	Part 4 (GC)n
*HLA-DRB1*13:02:01*	(GT)20,30,31	(GA)10-12CA(GA)3CA	(GA)6	(GC)2
*Patr-DRB1*03:07*	(GT)11-13		(GA)17-23	(GC)2
*HLA-DRB1*11:01:01*	(GT)22-26	(GA)5CA(GA)3CA	(GA)6	(GC)2
*Patr-DRB1*03:02*	(GT)23-28	(GA)5AA	(GA)6	(GC)4
*HLA-DRB3*01:01:02*	(GA)3(GT)13,14	(GA)9,10GGAA(GA)2CA(GA)3GG	GA	(GC)3
*Patr-DRB1*07:01*	(GT)2GGTT(GT)14	(GA)4GC	(GA)2	CCGC
*HLA-DRB1*07:01:01*	(GT)11	(GA)8GC	(GA)2	CCGC
*HLA-DRB1*15:01:01*	(GT)15-20	(GA)5-6CA(GA)4CA(GA)3GGAA	(GA)6	(GC)2
*Patr-DRB1*02:01*	GTGA(GT)19-22	(GA)12-14CA(GA)4CA(GA)3GGAA	(GA)6-7	(GC)2
*HLA-DRB1*01:03:01*	(GT)16	AAGAAA	(GA)4	(GC)3
*HLA-DRB1*01:01:01*	(GT)16,17	AAGAAA	(GA)4	(GC)3
*Hymo-DRB*W098:01*	(GT)6CT(GT)8,9		(GA)14	GC
*Hymo-DRB*W098:02*	GTGA(GT)5GA(GT)7	(GA)2(CA)2	(GA)4	GC
*Hymo-DRB*W099:01*	(GT)6,7CT(GT)13		(GA)11-13	GC
*Hymo-DRB1*04:01*	(GT)21-24	GA(CA)2	(GA)4	GC
*Hymo-DRB1*04:02*	(GT)19	GA(CA)2	(GA)4	GC
*Hymo-DRB1*04:04*	(GT)21	GA(CA)2	(GA)4	GC
*Hymo-DRB1*04:03*	(GT)12	(GA)2(CA)2	(GA)4	GC
*HLA-DRB1*04:01:01*	(GT)21-22		(GA)15,16	(GC)2
*HLA-DRB1*10:01:01*	(CT)2(GT)16		(GA)8	(GC)3
*Hymo-DRB*W100:01*	(GT)4GA(GT)2TT(GT)5	(GA)12GGAA	GA	GC
*Patr-DRB1*10:01*	(GT)9-10	(GA)11-12)CA(GA)4AA	(GA)5	(GC)3
*HLA-DRB5*01:01:01*	(GT)18-24	(GA)5-8GGAA(GA)4CA(GA)2GG	GA	(GC)3
*HLA-DRB5*01:02:01*	(GT)22	(GA)8GGAA(GA)4CA(GA)7GGAA(GA)4CA(GA)2GG	GA	(GC)3
*Patr-DRB5*03:10*	(GT)4GA(GT)7	(GA)10GGAA(GA)4CA(GA)2GG	GA	(GC)3
*Patr-DRB5*03:01*	(GT)4GA(GT)7	(GA)10GGAA(GA)4CA(GA)2GG	GA	(GC)3
*Patr-DRB5*03:06*	(GT)4GA(GT)7	(GA)10GGAA(GA)4CA(GA)2GG	GA	(GC)3
*Hymo-DRB*W096:01*	(GT)20	(GA)2(CA)2	(GA)4	GC
*Hymo-DRB*W096:02*	(GT)13	GA(CA)2	(GA)4	GC
*Hymo-DRB*W097:01*	GC(GT)20	GACA	(GA)7	GC
*Hymo-DRB*W095:01*	(GT)20-25	GG(CA)2	(GA)4	GC
*Hymo-DRB*W094:01*	(GT)18	GG(CA)2	(GA)4	GC
*Hymo-DRB*W094:02*	(GT)18,19	GG(CA)2	(GA)4	GC
*Patr-DRB6*01:08*	(GT)4	GAGGGCA(GG)2TC(GG)3GCAG	(GA)6	
*Patr-DRB6*03:05*	(GT)4	GGGAGGA(GG)3GTGGA(GG)2CGATAGG	(GA)8	
*HLA-DRB6*02:01*	(GT)13-24		(GA)11-14	
*HLA-DRB4*01:01:01*	GTAT(GT)9-11	(GA)9-14(CAGA)1,2GGAA	(GA)5	GC(GT)1,2(GC)1,2
*Patr-DRB4*01:04*	GTAT(GT)4-5	(GA)8-9(CAGA)2(GA)10CAGATGAA(GA)3AA	GA	GCGT(GC)2
*HLA-DRB7*01:01:01*	(GT)2TT(GT)3T	(GA)5(CA)6		
*Patr-DRB7*01:01*	(GT)2TT(GT)3T	(GA)6(CA)5		
